# The Relation of the Thoracic and Abdominal Walls to the Spinal Column, Considered with Reference to the Treatment of Antero-Posterior Curvatures1Read before the American Orthopedic Association, in Boston, Mass., September 17, 1889.

**Published:** 1890-02

**Authors:** Louis A. Weigel

**Affiliations:** Rochester, N. Y.; Orthopedic Surgeon to the Free Out-Patient Department of the Rochester City Hospital; 54 North Clinton Street


Buffalo Medical and Surgical Journal

VOL. XXIX. FEBRUARY, 1890. NO. 7.

### Original Communications.

### THE RELATION OF THE THORACIC AND ABDOMINAL WALLS TO THE SPINAL COLUMN, CONSIDERED WITH REFERENCE TO THE TREATMENT OF ANTERO-POSTERIOR CURVATURES.[1]

By LOUIS A. WEIGEL, M. D., Rochester, N. Y.

Orthopedic Surgeon to the Free Out-Patient Department of the Rochester City Hospital.

THE intimate anatomical relation which exists between the different component parts of the trunk, leads to the inference that there must necessarily be a correlation of physiological action. It is self-evident, therefore, that there cannot be a change of condition in any one part without affecting, secondarily, all others, to a greater or lesser degree.

I propose, in this paper, to briefly review the relation which the thoracic and abdominal walls bear to the spinal column, and endeavor to show that they assist materially in maintaining the upright position of the body; and must, therefore, be considered in the study of spinal curvatures

For a long time, the muscular system alone was considered the active force in the induction and maintenance of the various positions of the body. It is a recognized fact, however, that active muscular action, even when continued for a comparatively short time, induces fatigue, and results in diminution of power. It is, therefore, neces sary to view the function of the muscular system in an entirely different light. Active muscular contraction, undoubtedly, institutes changes of position and opposes a disturbance of equilibrium when the center of gravity of the body is displaced. It is also the active force which transfers the center of gravity to a point where an equilibrium may be established. But the ultimate effect of all muscular

1. Read before the American Orthopedic Association, in Boston, Mass., September 17, 1889.

action is simply to decrease the work imposed upon the muscular system to the greatest possible extent, thus allowing the physical forces to do their share of work.

With reference to the spinal column, it has been clearly demonstrated, by experiment, that the physical forces are sufficient to maintain the upright position, without the intervention of muscular action. This fact is also demonstrated, clinically, in cases of paralysis of the sacro-spinal muscles, in which muscular action is entirely eliminated. Meyer[1] relates an interesting case of this kind. The patient was a boy of fourteen years of age. When asked to pick up an object from the floor, he dropped forward to reach it; then, grasping the legs with his hands, he climbed up on them by raising alternately the sides of the trunk by contraction of the abdominal muscles, thus enabling him to grasp the leg somewhat higher up. When he reached the knees, he placed both hands on them and raised himself rapidly by a simultaneous extension of the knee- and hip-joints, at the same time throwing the trunk backwards, by forcibly extending the elbow-joints. He was then enabled to stand and maintain the upright posture.

In addition to this inherent tendency of the spine to remain erect, it is sustained by all the structures connected with it. In order to ascertain the exact relation the spine bore to other parts of the trunk, W. Parrow,[2] of Berlin, conducted a series of experiments on the cadaver. It was found by him that, when the pelvis was fixed in such a position as to allow the trunk to balance, the removal of the abdominal viscera was followed by an appreciable forward and downward movement of the seventh cervical vertebra. When the thoracic cavity was emptied, the spinal column moved upward and backward to a certain extent, but did not attain its primary position. In another experiment, simple incision of the abdominal walls was found to be sufficient to allow the vertebræ to sink downward and forward.

In the first experiment spoken of, it will be readily seen that, by the removal of the abdominal viscera, the thoracic organs are deprived of their natural support, and drag the column forward by their own weight. Thus, the center of gravity is displaced. This, however, may be restored by forcing the column back and increasing the normal posterior convexity of the dorsal segment, which will enable it to sustain the entire weight of the remaining portions of the trunk.

The effect of removing the thoracic viscera, after the abdominal cavity is emptied, is apparent. The weight of the trunk is lessened, so that the center of gravity is not displaced to any great extent. The spinal column, therefore, remains more nearly in its proper position.

1. Albert's Chirurgie, Bd. II., 1881, S. 33.

2. Virchow's Archiv., Bd. XXXI., 1864, p. 97.

The relation of the thorax to the spinal column is another point of interest. As stated by Parrow,[1] the first and tenth pair of ribs, with the vertebræ and sternum, form an elastic arch, which is strengthened by the interposition of other ribs. This increases its power of resistance to forces which tend to alter its shape. The anterior concavity of the dorsal curve is maintained by this costo-sternal arch, and the whole thorax forms an integral part of the spinal column. Anything which destroys or interferes with the integrity of the costo-sternal arch, must necessarily change the relative conditions of the spinal column and thorax.

It has been demonstrated that, when the integrity of the arch is destroyed, as, for instance, by removal of the sternum, the normal curve of the dorsal region becomes more extended, and forms a curve of greater radius; at the same time, the lumbar curve disappears entirely. This would seem to show that the superincumbent weight, or the simple force of gravity, is not sufficient to overcome the resistance to extension which the spinal column acquired by its connection with the costo-sternal arch.

The organs which this arch surrounds cannot, of course, be subjected to direct pressure, on account of the comparatively firm chest-walls. As Beely[2] well says, there cannot be an increase of the intra-thoracic pressure, in the sense that we speak of an increase of the intra-abdominal pressure; for, with every respiratory movement and opening of the glottis, an equalization of pressure would take place. The thoracic viscera, however, may be acted upon indirectly through the diaphragm; and in consequence of the intimate connection which exists between the ribs and vertebræ, the latter may be directly influenced by the whole thorax.

In the elaborate work of Parrow, who made a practical study of the physical conditions of the upright posture and the normal curvatures of the spinal column, upon the cadaver and the living subject, there are many other points of interest, which, however, cannot be enlarged upon at the present time.

Aside from anatomical and physiological facts, clinical evidence can be adduced to prove that the abdominal and thoracic walls assist in supporting the spinal column. Thus, it is a matter of common observation that when a patient is suffering from Potts's disease, the necessity of supporting himself on surrounding objects is not so urgent when sitting as when standing. In the sitting posture, the superincumbent weight of the head and upper part of the trunk

1. Loc. cit., p. 105.

2. Volkmann's Sammlung Klinischer Vorträge. No. 199, p. 11.

remains the same as in standing; but, in the former posture, the abdomen can be subjected to a certain amount of pressure against the pelvis; and thus, by forcing the viscera back and increasing the intra-abdominal pressure, act as a support to the weakened spine. Again, we often see patients suffering from Potts's disease make firm pressure over the abdomen, with both hands, during a paroxysm of pain; and frequently they experience the greatest amount of relief in the recumbent posture, by lying on the belly, hence instinctively assume this posture.

Bampfield[1] has called attention to the fact that, in antero-posterior curvature of the spine, the abdominal muscles cannot act in the same manner as when they are in a normal condition; that they cannot exert the necessary pressure upon the viscera and act as effectual antagonists to the diaphragm, which causes the difficulty of respiration and disturbs the activity of the intestinal tract.

Heather Bigg[2] also says:

> As a large portion of the abdominal organs are those dedicated to the function of digestion, it will easily be appreciated how curvature of the spine, by producing mechanical displacements of the organs, will give rise to a loss of alimentary tone, digestive derangements, and such constitutional disturbances as are dependent on ill or perverted nutrition; and it is not only a fact that such symptoms are accompaniments of spinal curvature, but also that they disappear under the mechanical treatment that is employed to cure the curvature itself.

A short, grunting and suppressed respiration is one of the prominent symptoms of Potts's disease. This symptom has been ascribed to an effort on the part of the patient to prevent motion in the spinal column. The general muscular rigidity of the trunk present in these cases is undoubtedly an effort on the part of the patient to make a muscular splint, as Sayre says; but the fact that by placing the child across the lap and making gradual extension, free and easy respiration is secured, cannot be attributed entirely, in my opinion, to the possible relief of the nerves from pressure. For, in some cases in which a diminution of the curvature does not take place and is not to be expected, the respiration becomes tranquil and regular after the application of any jacket which secures a uniform support of the trunk.

In this connection the history of the first case in which Sayre applied his jacket, is of interest. You will remember that the patient was a boy who was suffering from disease of the three last dorsal and first lumbar vertebræ. Sayre applied a jacket over a well-fitting shirt, without resorting to the use of the so-called dinner-pad. After it was applied he was fearful lest it might interfere too much with

1. Quoted by Beely, Op. cit.
2. Spinal curvature. London. 1882. P. 107.

respiration, and, therefore, cut the jacket through from top to bottom, thus permitting a more complete expansion of the chest. The patient was at once less comfortable than before the jacket was cut. Sayre ascribes the immediate good effect of the dressing to the rest of the diseased parts secured thereby. But, with a disease in the dorsal region, absolute rest certainly cannot be secured, because every respiratory effort would necessarily produce motion at the diseased part. It is more reasonable to ascribe the efficiency of the dressing to the equable support of the spine, and firm pressure upon the abdomen.

The fact that a curvature sometimes increases in extent in spite of the jacket, when the disease is located in the dorsal region, is a proof that the jacket does not support the spine as a splint does a fracture. Sayre recognizes this fact by advising the use of a jury-mast in all cases where the disease is located above the mid-dorsal region. The upper portion of the dorsal region is less under the direct influence of the intra-abdominal pressure and does not receive the direct support of the abdominal walls; hence, the weight of the parts above exerts an injurious pressure upon the weakened spot and produces the marked curvature. This difficulty is obviated, to a certain extent, by the jury-mast.

A word with reference to the dinner-pad. The use of a dinner-pad is advised for the purpose of leaving room for meals. It is apparent, however, that this is of little practical importance, for, unless a very large pad is used, the intended object will not be accomplished. Leaving a large hollow space over the abdomen, must necessarily interfere with the stability and efficiency of the jacket. In the history of Sayre's first case, as already stated, no mention is made of the dinner-pad, and none was probably used, as it is distinctly stated that plaster-of-Paris bandages were applied around the entire trunk over a close-fitting shirt. For the past few years, I have discarded entirely the use of this pad, and have seen no harm come from the omission. It has been my experience, furthermore, that patients express themselves as more comfortable when the support is applied firmly and in direct contact with the abdomen.

In the treatment of spinal affections, mechanical problems present themselves that are sometimes exceedingly difficult. The various segments of the spinal column differ not only in their relation to each other, but also in their relation to surrounding parts, which modify their function in health and demand consideration in disease. It is only by a careful study of *all* existing conditions, from an anatomical, physiological, and mechanical standpoint, that we can intelligently and successfully treat antero-posterior curvatures of the spine.

54 North Clinton Street.

